# Acceptability and Feasibility of Pharmacy-Based Delivery of Pre-Exposure Prophylaxis in Kenya: A Qualitative Study of Client and Provider Perspectives

**DOI:** 10.1007/s10461-021-03229-5

**Published:** 2021-04-07

**Authors:** Stephanie D. Roche, Njeri Wairimu, Peter Mogere, Kevin Kamolloh, Josephine Odoyo, Zachary A. Kwena, Elizabeth A. Bukusi, Kenneth Ngure, Jared M. Baeten, Katrina F. Ortblad

**Affiliations:** 1 Department of Global Health, University of Washington, 325 Ninth Avenue, Seattle, Washington 98104 USA; 2 Partners in Health and Research Development, Nairobi, Kenya; 3 Center for Microbiology Research, Kenya Medical Research Institute, Kisumu, Kenya; 4 Department of Obstetrics and Gynecology, University of Washington, Seattle, USA; 5 Department of Community Health, Jomo Kenyatta University of Agriculture and Technology, Nairobi, Kenya; 6 Department of Medicine, University of Washington, Seattle, USA; 7 Department of Epidemiology, University of Washington, Seattle, USA

**Keywords:** Pre-exposure prophylaxis, Implementation science, Kenya, Differentiated care, HIV prevention

## Abstract

As countries scale up pre-exposure prophylaxis (PrEP) for HIV prevention, diverse PrEP delivery models are needed to expand access to populations at HIV risk that are unwilling or unable to access clinic-based PrEP care. To identify factors that may influence implementation of retail pharmacy-based PrEP delivery in Kenya, we conducted in-depth interviews with 40 pharmacy clients, 16 pharmacy providers, 16 PrEP clients, and 10 PrEP providers from two provinces. Most participants expressed strong support for expanding PrEP to retail pharmacies, though conditioned their acceptance on assurances that care would be private, respectful, safe, and affordable. Participant-reported determinants of feasibility centered primarily on ensuring that the intervention is compatible with retail pharmacy operations (e.g., staffing levels, documentation requirements). Future research is needed to develop and test tailored packages of implementation strategies that are most effective at integrating PrEP delivery into routine pharmacy practice in Kenya and other high HIV prevalence settings.

**Supplementary Information:**

The online version contains supplementary material available at 10.1007/s10461-021-03229-5.

## RESUMEN

Ya que varios países están ampliando sus programas de profilaxis previa a la exposición al VIH (PrEP, por sus siglas en inglés), se necesitan modelos diversos para ampliar el acceso a poblaciones que no están dispuestos a acceder, o que no pueden acceder, a los servicios de PrEP que se ofrecen en las instalaciones de salud. Para identificar los factores que pueden influir en la prestación de servicios de PrEP a través de farmacias privadas, realizamos entrevistas en profundidad con 40 clientes de farmacia, 16 proveedores de servicios de farmacia, 16 usuarios de PrEP, y 10 proveedores de PrEP. La mayoría de los participantes manifestó su firme apoyo a la propuesta de expandir la provisión de PrEP a las farmacias privadas, aunque condicionó su aceptación a la garantía de que la atención sea privada, respetuosa, segura, y asequible. Según los participantes, los factores determinantes de la viabilidad de ofrecer PrEP en las farmacias privadas se centran en asegurar de que la intervención sea compatible con las operaciones de las farmacias privadas (p. ej., el número de personal, los requisitos de documentación). Se necesitan investigaciones adicionales para desarrollar y evaluar diferentes paquetes de estrategias de implementación para descubrir cuáles son los más eficaces para integrar los servicios de PrEP en la prestación rutinaria de servicios de farmacia tanto en Kenia como en otros lugares de alta prevalencia del VIH.

## Introduction

In the nearly 10 years since clinical trials confirmed the safety and efficacy of pre-exposure prophylaxis (PrEP) for HIV prevention, diverse PrEP delivery models have been implemented around the world, primarily in high-income settings [1, 2]. In the U.S., home to the world’s largest number of PrEP users, PrEP is delivered through a variety of clinic-based, pharmacy-based, and telehealth models [3–5]. In sub-Saharan Africa, where PrEP delivery remains largely confined to clinics, the need for differentiated PrEP delivery models that reach populations at HIV risk and reduce burdens on health systems remains high [6]. Even in Kenya, which has the second highest number of PrEP users in the world, findings from open label, demonstration, and implementation projects have found that client desire for public clinic-based PrEP delivery is tempered by concerns about stigma, opportunity costs, and limited privacy [7–10]. Moreover, clinic-based PrEP may not reach Kenyans at HIV risk who do not regularly frequent healthcare clinics [11].

In 2017, the Kenyan Ministry of Health (MOH) released a 5-year plan for increasing access to PrEP [12], which includes scaling up PrEP delivery to additional public HIV clinics and scaling out PrEP delivery to other venues [13], including retail pharmacies. With approximately 5,800 registered outlets nationwide [14], retail pharmacies represent a promising platform for increasing PrEP accessibility and coverage in Kenya. Previous studies have found that Kenyans seeking treatment for minor ailments and preventive care often resort first to retail pharmacies, even when the desired product or service is available for free at public clinics [15–18]. In recent years, standalone pharmacy-based PrEP delivery programs have been successfully implemented in select parts of the U.S. (e.g., Seattle, San Francisco, Miami, Denver, St. Louis, Jackson, and Omaha) [5, 19–24].

The acceptability and feasibility of pharmacy-based PrEP delivery has not been studied in a sub-Saharan African country. We conducted qualitative formative research to inform the design and implementation of a pharmacy PrEP care pathway in Kenya. Specifically, we aimed to understand perceptions among pharmacy PrEP stakeholders about the proposed intervention, including factors that may influence their ability or willingness to uptake or deliver PrEP at retail pharmacies.

## Methods

### Participants

We purposefully sampled pharmacy clients, pharmacy providers, PrEP clients, and PrEP providers from two counties: Kiambu County in central Kenya and Kisumu County in western Kenya, where HIV prevalence is 4% and 16%, respectively. Trained research assistants (RAs) contacted providers to describe the research and invite them to participate. RAs approached potential client participants as they exited select retail pharmacies or HIV clinics in a variety of socioeconomic settings and scheduled interested, eligible individuals for an interview.

Eligible pharmacy clients self-reported being HIV negative and were assessed as at HIV risk using Kenya’s Rapid Assessment and Screening Tool (RAST) [25]. Eligible pharmacy providers were licensed pharmacists or pharmaceutical technologists employed at a registered retail pharmacy. Eligible PrEP clients and PrEP providers obtained or delivered PrEP services at a public HIV clinic. All participants were 18 years or older.

### Data Collection

We developed semi-structured interview guides to gather data on five domains, including health-seeking behaviors, experiences taking PrEP, delivery practices, anticipated benefits and drawbacks of pharmacy PrEP, and anticipated barriers and facilitators to pharmacy PrEP. We used the Consolidated Framework for Implementation Research (CFIR) [26], a meta-theoretical framework of constructs hypothesized to predict, moderate, or “drive” implementation outcomes, such as acceptability and feasibility [27, 28], to frame questions related to barriers and facilitators. We captured participant demographics via close-ended questions. We translated, back-translated, and pilot tested the guides, and excluded pilot interviews from our analytic data.

From October 2019 to April 2020, Kenyan RAs conducted individual interviews with participants in their preferred language: English, Kiswahili, or Dholuo. Interviews took place in a private room at the pharmacy, HIV clinic, or study research office, typically lasted 51 min (interquartile range [IQR]: 40–63 min), and were audio-recorded. After each interview, RAs transcribed the recording and, if applicable, simultaneously translated it to English.

Author SDR checked the quality of each RA’s first three transcripts and performed random spot-checks on all remaining transcripts. For non-English interviews, an RA fluent in the language performed the quality check.

### Data Analysis

A subset of the research team (SDR, KFO, and NW) with university-level training in qualitative methodology analyzed the interview data in Dedoose version 8.0.35 (SocioCultural Research Consultants, LLC, Los Angeles, USA) using conventional content analysis [29], an inductive approach that involves systematic review, reduction, and interpretation of the data [30]. These authors developed, tested, and refined a codebook based on a comparison of independently coded transcripts. SDR and NW coded the remaining transcripts and resolved disagreements via consensus. We conducted second cycle pattern coding to develop a coherent meta-synthesis of the data [31, 32], dropping redundant or irrelevant codes, merging similar codes, and subdividing codes encompassing distinct concepts. We organized remaining codes into “meta-codes” representing determinants of acceptability and feasibility and mapped these onto the CFIR framework. Using RStudio (RStudio Team, 2016), we assessed descriptive statistics of participants’ demographic information, including description of central tendency as frequency and percent or median and interquartile range, as appropriate.

### Ethics

The institutional review boards of the University of Washington and the Kenya Medical Research Institute approved this study. We obtained written informed consent from all participants. The Electronic Supplementary Material (Sect. 1) contains additional details about our methodology presented using the Consolidated Criteria for Reporting Qualitative Research (COREQ) checklist.

## Results

We interviewed 82 individuals: 40 pharmacy clients, 16 pharmacy providers, 16 PrEP clients, and 10 PrEP providers (Table 1). Of the eligible 49 pharmacy clients invited to interview, 9 declined due to time constraints (82% participation). All remaining groups had 100% participation.

**Table 1:** Participant demographics

Variable	Clients		Providers	
	PrEP (n = 16)	Pharmacy (n = 40)	PrEP (n = 10)	Pharmacy (n = 16)
Age in years^a^	28 (23–29)	25 (22–28)	37 (35–40)	33 (27–35)
Female	9 (56%)	20 (50%)	7 (70%)	7 (44%)
Occupation				
Unemployed	2 (13%)	5 (13%)	–	–
Student	0 (0%)	4 (10%)	–	–
Business/sales	5 (31%)	12 (30%)	–	–
Hospitality/service industry	8 (50%)	3 (8%)	–	–
Medical doctor	0 (0%)	0 (0%)	2 (20%)	0 (0%)
Clinical officer	0 (0%)	0 (0%)	3 (30%)	0 (0%)
Nurse	0 (0%)	0 (0%)	2 (20%)	0 (0%)
HIV testing services (HTS) counselor	0 (0%)	1 (3%)	2 (20%)	0 (0%)
Pharmacist	0 (0%)	1 (3%)	0 (0%)	1 (6%)
Pharmaceutical technologist	0 (0%)	1 (3%)	0 (0%)	15 (94%)
Other	1 (6%)	13 (33%)	1 (10%)	0 (0%)
Recruitment location^b^				
Urban area - informal settlement	4 (25%)	7 (18%)	1 (10%)	6 (38%)
Urban area - non-informal settlement	9 (56%)	14 (35%)	2 (20%)	3 (19%)
Peri-urban area	0 (0%)	9 (23%)	4 (40%)	4 (25%)
Rural area	3 (19%)	10 (25%)	3 (30%)	3 (19%)
Educational attainment^c^				
Less than high school	6 (38%)	1 (3%)	–	–
High school graduate	5 (31%)	9 (23%)	–	–
Some college or college certificate/diploma	3 (19%)	18 (45%)	–	–
Some university or university degree	2 (12%)	12 (30%)	–	–
Married	9 (56%)	6 (15%)	–	–
Number of children^a^	1 (1–2)	0 (0–1)	–	–
Monthly household income in Kenyan shillings^a,d^	20,000 (11,750–28,750)	30,000 (20,000–55,000)	–	–
Months on PrEP^a^	11 (7–24)	–	–	–
To reach PrEP clinic				
Travel time, in minutes^a^	45 (30–60)	–	–	–
Travel cost, in Kenyan shillings^a,e^	65 (30–100)	–	–	–
To reach preferred retail pharmacy				
Travel time, in minutes^a^	10 (5–30)	6 (5–10)	–	–
Travel cost, in Kenyan shillings^a,f^	0 (0–30)	0 (0–0)	–	–

^a^Presented as median (interquartile range)

^b^Location of healthcare clinic or pharmacy where provider works or where the client obtains pharmacy or PrEP services

^c^College certificates, college diplomas, and university degrees generally take a minimum of 1, 2, and 4 years, respectively, to complete

^d^Approximately USD $190 ($110–270) for PrEP clients and $280 ($190–510) for pharmacy clients

^e^Approximately USD $0.60 ($0.28–0.93)

^f^Approximately USD $0 ($0–0.28) for PrEP clients. Most (45/56) client participants reported a transportation cost of 0 KES because they walk to their preferred retail pharmacy

The median age of participants was 27 years (IQR 23–35 years) and about half were female (43/82). The median time PrEP clients used PrEP was 11 months (IQR 7–24 months). The sample contained roughly equal numbers of medical doctors, clinical officers, nurses, and HIV testing service (HTS) counselors. With the exception of one pharmacist, all pharmacy providers were pharmaceutical technologists, and 75% (12/16) were pharmacy owners.

**Fig. 1:**
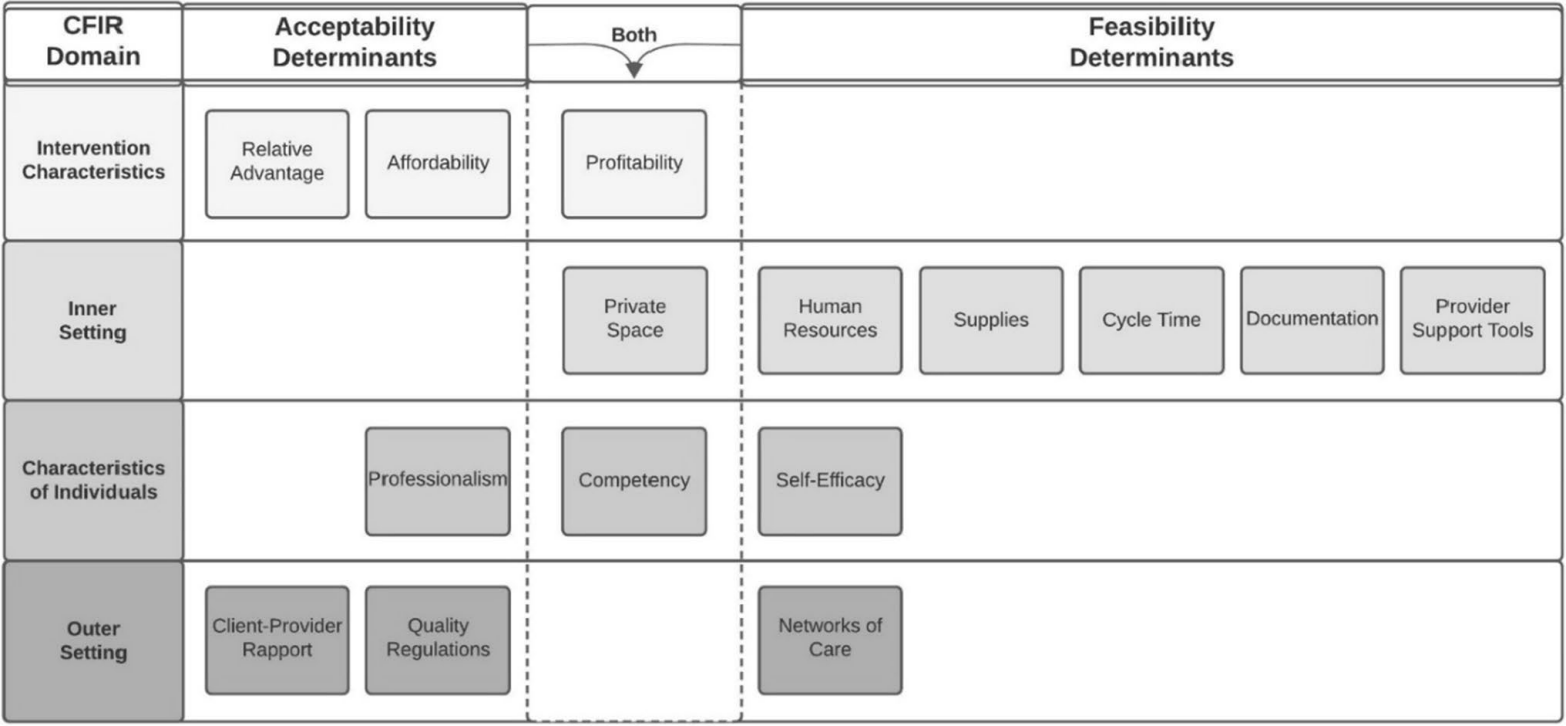
Determinants of pharmacy-based PrEP delivery acceptability and feasibility. Factors influencing the acceptability and feasibility of pharmacy-based PrEP delivery, organized by domains of the Consolidated Framework for Implementation Research (CFIR)

Below, we describe participant-reported relative advantages of pharmacy-based versus clinic-based PrEP delivery (Table 2) and determinants of acceptability and feasibility, organized by CFIR domain (Fig. 1). Throughout, we reference illustrative quotes (Tables 3 and 4) by number and letter (e.g., “Quote 2A” refers to quote A in Table 2). The Electronic Supplementary Material contains additional results, including specific recommendations proposed by participants.

**Table 2:** Anticipated relative advantages of pharmacy-based versus clinic-based PrEP delivery

Advantage	Beneficiary	Illustrative quote	
*Proximal advantages*			
Convenience	PrEP clients	(A)	*Ubiquity*: “The beauty is that pharmacies are all over. I could be walking [back to work] from lunch and decide to pass by a pharmacy [to] get my PrEP.” *(Kiambu PrEP Provider 10)*
		(B)	*Fast service time*: “[At the pharmacy,] you can take like 20 minutes, and you are out, rather than going to queue for the whole day [at a hospital].” *(Kisumu Pharmacy Client 6)*
		(C)	*Long opening hours*: “You can go to the pharmacy any time because some even work up to midnight. So it [getting PrEP at the pharmacy] wouldn’t disrupt you from your work, unlike the hospital, which closes at 5 pm.” *(Kiambu PrEP Client 5)*
		(D)	*Open weekends*: “In the hospital, you might go [to get PrEP] on a Friday, and they tell you, ‘No, we stopped giving [PrEP] at 1 PM. Come back on Monday’…[But] at the chemist, you will get the drugs even if it is on a Sunday. There’s no limit on the days.” *(Kiambu PrEP Client 8)*
		(E)	*Low/no transportation cost*: “[With pharmacy PrEP], you don’t have to travel to the hospital to get PrEP. It is just readily available near where you live, so you would be able to save the [transportation] cost.” *(Kiambu PrEP Provider 4)*
Privacy	PrEP clients	(F)	“There are people who would be afraid of being seen at the [HIV] clinic [when getting PrEP] … The advantage [of pharmacy PrEP] is that there is no stigma. No one knows which medicine you are getting there [at the pharmacy].” *(Kiambu PrEP Client 2)*
		(G)	*Choice of location/care provider*: “[I would] prefer going to a person [pharmacy provider] that doesn’t know me … So I would go to a distant chemist where I will be comfortable.” *(Kiambu PrEP Client 8)*
Autonomy	PrEP clients	(H)	*Choice of care timing*: “Here [with pharmacy PrEP] is also what we call ‘time efficiency’ because if you decide to walk to this pharmacy and see that it is crowded and you are in a hurry, you have the authority to move to the next pharmacy.” *(Kiambu PrEP provider 10)*
Profit	Pharmacies	(I)	“[PrEP] can be a source of income to the pharmacy.” *(Kisumu Pharmacy Provider 6)*
*Distal advantages*			
Expanded access	PrEP clients	(J)	“[Pharmacy PrEP] could be a better option, especially for those [clients] who cannot reach the bigger hospitals where PrEP is available.” *(Kisumu Pharmacy Client 13)*
Increased uptake	PrEP clients	(K)	“I think it [pharmacy PrEP] would improve PrEP uptake in our country because it would remove the barriers [to clinic-based PrEP], especially for adolescents and male populations who feel more comfortable going [to pharmacies] to buy [PrEP].” *(Kiambu PrEP Provider 10)*
Increased adherence	PrEP clients	(L)	“[Pharmacy PrEP] would improve adherence because sometimes people run out of PrEP and feel lazy to go all the way to the hospital or they don’t have the [transportation] fare, so they delay. But if it is [available] at the chemist, you will just go and buy because it is near.” *(Kiambu PrEP Client 12)*
		(M)	“Sometimes they [PrEP clients] forget their medication and they’ve traveled far … [It would be great] if we could tell them, ‘You can walk to a nearby chemist’ and they can continue taking the medication.” *(Kiambu PrEP Provider 10)*
Reduced HIV incidence	Society	(N)	“If it [PrEP] is made available in pharmacies, it would really reduce the rate of [HIV] transmission … We are struggling to achieve 90-90-90 [targets] … [Pharmacy PrEP] will encourage more people to test and know their [HIV] status.” *(Kiambu PrEP Provider 3)*
Decongested clinics	PrEP clients, providers	(O)	“If we get pharmacy-based PrEP delivery services, it will relieve the work of this [PrEP] clinic very much. And it is to the advantage of those other [PrEP] patients that need a lot of time [with clinic-based PrEP providers].” *(Kiambu PrEP Provider 8)*

**Table 3:** Determinants of acceptability of pharmacy-based PrEP delivery, organized by components of the Consolidated Framework for Implementation Research

CFIR domain	CFIR construct: specific determinant		Illustrative quote
Intervention characteristics	Relative advantage:^a^ Relative advantage	(A)	“If those who feel stigmatized queuing here [at the hospital] can pick it [PrEP] from somewhere else, it will decongest [public PrEP clinics].” *(Kiambu PrEP Provider 3)*
	Cost: Affordability	(B)	“[Whether I will get PrEP at a pharmacy] will depend on the cost. I may find that I have run out of drugs and I don’t have money. So that may be a challenge.” *(Kiambu PrEP Client 10)*
	Cost: Profitability	(C)	“This is a business also … so you can’t expect someone to handle [PrEP] clients unless they bring money.” *(Kiambu Pharmacy Provider 4)*
Inner setting	Available resources: Privacy	(D)	“[The HIV testing and PrEP counseling] must be done in a private room in the chemist … Clients want to feel relaxed and secure.” *(Kiambu PrEP Client 14)*
Characteristics of individuals	Knowledge and beliefs: Competency	(E)	“[Pharmacy PrEP could work] as long as whoever is mandated [to deliver PrEP at the pharmacy] is well-trained and they understand the importance of PrEP and what it does, what is going to happen if it is misused, the benefits, and all that.” *(Kiambu PrEP Provider 4)*
	Other personal attributes: Professionalism - Ethics	(F)	*Ethics*:“[Pharmacy PrEP acceptability] is all about the [ability of the] pharmacist at the chemist to keep clients’ confidentiality because PrEP has stigma.” *(Kiambu PrEP Client 14)*
	- Respect	(G)	*Respect*: “[Pharmacy PrEP providers] should not have a judgmental attitude… If you tell them you had unprotected sex recently … [they need to] be understanding.” *(Kiambu Pharmacy Client 12)*
	- Integrity	(H)	*Integrity*: “Here [at the hospital] I will follow the rules and say [to a client], ‘If you are not [HIV] tested, I will not issue the [PrEP] drugs.’ But in the pharmacy … they may be interested in the money over the testing.” *(Kiambu PrEP Provider 9)*
Outer setting	Patient needs and resources: Rapport	(I)	“[Pharmacy PrEP providers] should have good relationships with their clients … [The provider] there at the pharmacy [I go to], we are like friends. He knows my medical history.” *(Kisumu Pharmacy Client 8)*
		(J)	“[Some clients] will not feel comfortable … if [the pharmacy PrEP provider] knows them personally. So they will prefer to pick them [PrEP drugs] from another chemist.” *(Kiambu Pharmacy Client 10)*
	External policies and incentives: Quality regulations	(K)	“Clients should be protected … [and] assured that they will get good health services … Chemists that offer PrEP should meet some minimum standards to handle patients.” *(Kiambu PrEP Client 14)*

^a^For additional results on relative advantages, see Table 2

**Table 4:** Determinants of feasibility of pharmacy-based PrEP delivery, organized by components of the Consolidated Framework for Implementation Research

CFIR domain	CFIR construct: Specific determinant	Illustrative quote	
Intervention characteristics	Cost: Profitability	(A)	“[To make PrEP financially sustainable for pharmacies to deliver,] there must be a fee [you can charge] … Then you’ll be able to afford the space, the record-keeping, the [time and resources for client] follow-up.” *(Kiambu Pharmacy Provider 6)*
Inner setting	Available resources: Space	(B)	“Maybe when I open another pharmacy with more space, [I’ll deliver PrEP] … But in this pharmacy, I can’t because I only have a dispensing area … You can’t do counseling when other people are standing there [at the pharmacy counter] … You need privacy.” *(Kiambu Pharmacy Provider 6)*
	Available resources: Human resources	(C)	“The challenge can be if the person working [at the pharmacy] is only one … because if you are only one person who is counselling and also selling the drugs, then it becomes a challenge.” *(Kisumu Pharmacy Provider 4)*
	Available resources: Supplies	(D)	“[One potential challenge] is you might run out of stock of PrEP.” *(Kisumu Pharmacy Provider 2)*
	Compatibility: Cycle time	(E)	“Most pharmacies are very busy. [Typically,] you only give a client maybe 2 to 5 minutes. But [initiating a client on PrEP] … will take time—maybe around 30 minutes. Giving one client 30 minutes to 1 hours means you will serve less clients.” *(Kiambu Pharmacy Provider 7)*
	Compatibility: Documentation	(F)	“For DDA [drugs specified in the Dangerous Drugs Act], the Pharmacy and Poisons Board already requires us to keep a register with the client’s name, age, what is prescribed, number of pills, and the date … [For pharmacy PrEP,] we can keep a similar record of how we gave them PrEP.” *(Kiambu Pharmacy Provider 6)*
	Access to knowledge and information: Provider support tools	(G)	“[PrEP providers] have a checklist for [assessing] PrEP eligibility: the RAST [Rapid Assessment Screening Tool]. They [pharmacy PrEP providers] can use that one, too.” *(Kiambu PrEP Provider 7)*
Characteristics of individuals	Knowledge & Beliefs: Competency	(H)	“We [pharmacy providers] need information that will help in identifying eligible clients [for PrEP]. We need to get detailed information about side effects, how to manage, which ones to refer.” *(Kiambu Pharmacy Provider 3)*
	Self-efficacy: Self-efficacy	(I)	“[Say] somebody comes for HIV testing…and you did not give enough or adequate counseling, and then you hear the following day somebody has hanged himself or herself … I would be a bit worried [to do HIV testing and counseling].” *(Kiambu Pharmacy Provider 5)*
Outer setting	Cosmopolitanism: Networks of care	(J)	“It is important sometimes to consult further and make referrals where necessary. [For pharmacy PrEP,] we need a system that is integrated with the pharmacies that are offering PrEP so that they are networked and there are contacts.” *(Kisumu Pharmacy Provider 6)*

### THEME 1: Stakeholders are interested in trying pharmacy-based PrEP delivery and anticipate several short- and long-term advantages over clinic-based PrEP delivery

Nearly all participants said they would be interested in engaging in pharmacy PrEP, with some noting that demand for it already exists. Unprompted, about half (7/16) of pharmacy providers reported that clients routinely ask for PrEP at their pharmacy, and two such providers described occasionally acquiring PrEP for clients who request it. Similarly, two clients reported seeking PrEP at a pharmacy, one successfully. This client, however, later learned that the pharmacy provider’s instructions for use (which were to only take PrEP on days she anticipated having sexual intercourse) were inaccurate.

#### Proximal Advantages

##### Convenience

Citing the ubiquity of retail pharmacies, PrEP and pharmacy clients anticipated that pharmacy PrEP would save them travel time and fare, as most lived within walking distance of their preferred retail pharmacy (**Quote 2A**). PrEP clients reported a lower median travel time and fare to reach their preferred pharmacy than to reach their current PrEP clinic: 10 min (IQR: 5–30 min) and 0 Kenyan shillings (KSH) (IQR: 0–30 KSH) versus 45 min (IQR: 30–60 min) and 65 KSH (30–100 KSH). Clients also anticipated that wait time for PrEP at pharmacies would be shorter than that at clinics. The median time PrEP clients reported waiting to see a PrEP provider at public HIV clinics was 30 min (IQR: 20–75 min) (**Quote 2B**).

Many participants stated that pharmacy-based PrEP delivery would be more convenient than clinic-based PrEP delivery because retail pharmacies have longer opening hours (**Quote 2C**). According to provider responses about their own workplaces, pharmacies typically operate not only more days per week (7 days, IQR: 6–7 days) than public HIV clinics (5 days, IQR: 5–5 days), but also more hours per day (13 h, IQR: 12–13 h) than public HIV clinics (8 h, IQR: 8–8 h) (**Quote 2D**). Participants equated longer opening hours with fewer opportunity costs, such as having to take time off from work to get PrEP (**Quote 2E**).

##### Privacy

Participants, especially PrEP providers and clients, anticipated that pharmacy PrEP would circumvent stigma clients often face when accessing PrEP in HIV clinics. For example, many PrEP clients described fearing that friends, family members, or neighbors would see them at the HIV clinic and mistakenly think they are living with HIV. Noting that retail pharmacies offer a wide variety of products at the same counter, participants felt that clients would be able to obtain PrEP there discreetly without other customers knowing the reason for their visit (**Quote 2F**).

##### Autonomy

Reporting that the availability of clinic-based PrEP is largely restricted to select public HIV clinics, participants imagined that pharmacy PrEP (if expanded to numerous retail pharmacies) would give clients greater choice around when and where to seek PrEP care (**Quotes 2G** and **2H**).

##### Profit

Pharmacy providers viewed pharmacy PrEP as a potential source of profit (**Quote 2I**).

#### Distal Advantages

##### Reduced HIV Incidence

Participants from all groups felt that pharmacy PrEP would address client barriers to clinic-based PrEP delivery and, in turn, lead to more individuals accessing, initiating, and adhering to PrEP. They felt that these shifts in PrEP use would ultimately contribute to reductions in population-level HIV incidence (**Quotes 2J-2N**). PrEP providers further noted that diverting some PrEP clients to pharmacies may help decongest public HIV clinics and enable PrEP providers to spend more time on cases requiring a higher level of PrEP expertise (e.g., serodiscordant couples) (**Quote 2O**).

### THEME 2: The acceptability of pharmacy-based PrEP delivery hinges on meeting stakeholder expectations for quality of care

Most participants indicated they would find pharmacy PrEP acceptable so long as the quality of PrEP care delivered in pharmacies was on par with, or exceeded that, typically offered in clinics.

#### Intervention Characteristics

In addition to relative advantages, participants reported that the acceptability of pharmacy PrEP would depend on its affordability to clients and profitability for pharmacies (**Quotes 3A-3C**). Acknowledging that not all individuals at HIV risk will have the means to purchase PrEP, most clients expressed a desire for the MOH to collaborate with retail pharmacies to make pharmacy PrEP available at no or low cost to clients. Pharmacy providers similarly expressed a desire for the MOH to subsidize the cost of PrEP delivery so they could support this public health initiative.

#### Inner Setting

Nearly all participants anticipated that pharmacy PrEP would only be acceptable to clients if it took place in a private area of the pharmacy. Participants were particularly adamant that HIV testing and counseling, and any discussions about sexual activity, would need to occur in a separate counseling room out of earshot of other pharmacy clients (**Quote 3D**).

#### Characteristics of Providers

Participants identified provider competency and professionalism as determinants of acceptability. Both clients and providers stated that they would only find pharmacy PrEP agreeable insofar as they felt confident that pharmacy PrEP providers were not “quacks” but possessed adequate PrEP knowledge and skills (**Quote 3E**). Noting that some components of PrEP delivery (e.g., HIV testing and counseling) are outside of the traditional scope of practice for pharmacy providers in Kenya, providers stressed that pharmacy PrEP providers would need additional training. Clients also explained that their acceptance of pharmacy PrEP would depend, in part, on pharmacy providers’ ability to maintain client confidentiality, treat clients with respect (e.g., not judge clients’ sexual behaviors), and prioritize clients’ well-being over profit-making (**Quotes 3F-3H**).

#### Outer Setting

With respect to client-provider rapport, some clients anticipated wanting to get PrEP from a provider with whom they have an ongoing, close relationship (**Quote 3I**). Others stated that they would purposely seek PrEP from a provider who did not know them (**Quote 3J**). Participants found both options viable if pharmacies offering PrEP were spread across different localities.

Many participants perceived retail pharmacies to be less strictly regulated than public clinics and conditioned their acceptance of pharmacy PrEP on the existence of regulatory policy (e.g., quality standards) and oversight (e.g., audits). Both groups of clients and PrEP providers alike viewed the involvement of authorities, such as the national drug regulatory agency of Kenya, as key to ensuring clients receive safe and appropriate care (**Quote 3K**).

### THEME 3: For pharmacy-based PrEP delivery to be feasible, retail pharmacies may need to adapt their operations

#### Intervention Characteristics and Inner Setting

Pharmacy providers stressed that pharmacy PrEP would not be feasible to deliver in the absence of a private counseling room, sufficient staff, and consistent access to supplies (**Quotes 4A-D**). All pharmacy providers reported that their current daily workload was “manageable” or “very manageable” and that, during a typical day at their pharmacy, two providers (IQR: 1–2 providers) attended to roughly 45 clients (IQR: 34–100 clients), spending about 5 min (IQR: 5–10 min) with each. When asked the maximum amount of time they thought they could spend attending to a PrEP client, pharmacy providers’ median response was 30 min (IQR: 10–30 min). Most imagined that they would need to hire additional staff to carry out PrEP delivery, especially if the amount of time they spend serving a client (i.e., cycle time) is substantially higher for PrEP clients (**Quote 4E**). These providers noted that spending additional time with PrEP clients would only be feasible if the PrEP client volume and profit margin made it economically worthwhile. Profitability also emerged in discussions of supplies, such as HIV testing kits, with pharmacy providers reiterating their desire for government subsidies to offset these costs. Most pharmacy providers reported that if the MOH provided them with PrEP drugs for free, they would only charge the client a fee to cover the cost of drug dispensing, storage, and disposal.

Some PrEP providers noted that, if subject to the same reporting requirements as PrEP clinics, retail pharmacies may need to install systems for documenting PrEP care and tracking commodities (**Quote 4F**). Pharmacy providers, for their part, described using a wide variety of record-keeping practices, primarily to monitor inventory, but identified some standard documentation they currently perform (e.g., for prescription opioids) as a potential foundation onto which pharmacy PrEP documentation could be added.

#### Characteristics of Providers

Although most pharmacy providers stated that they routinely counsel clients, assess adherence, and monitor for side effects, they anticipated that pharmacy providers would require additional training to deliver PrEP, especially on pharmacovigilance, adherence counseling, and HIV testing and counseling (**Quotes 4H** and **4I**). Pharmacy providers typically felt that a 5-day (IQR: 3–5-day) training would suffice. Both pharmacy and PrEP providers thought trained pharmacy providers would be capable of safely prescribing PrEP to new clients. They further noted that provider support tools, such as the aforementioned RAST tool for assessing HIV risk and PrEP eligibility, could advance pharmacy providers’ competency and enhance their sense of self-efficacy (**Quote 4G**).

#### Outer Setting

Participants from both provider groups universally agreed that a care network through which pharmacy providers could consult PrEP experts and refer complex cases would increase the feasibility of pharmacy-based PrEP delivery (**Quote 4J**). Providers varied in their preferences for communication content, frequency, and platform, with some noting the need for an inter-provider collaboration protocol. Pharmacy providers reported that, currently, they seldom interact with prescribing clinicians, except to occasionally confirm an unusual dosage or request permission to alter a prescription for an out-of-stock medication.

## Discussion

Kenyan stakeholders found the concept of pharmacy PrEP delivery to be acceptable and conditioned their acceptance on assurances that care would be private, respectful, safe, and affordable. Similar to their counterparts in high-income settings [33–35], pharmacy and PrEP providers in our study were open to task-shifting PrEP delivery to retail pharmacy providers, though did identify factors that could reduce the feasibility of pharmacy-based PrEP delivery (e.g., insufficient provider time). Overall, most participants considered retail pharmacies an ideal venue for reaching individuals at HIV risk who are unable or unwilling to obtain PrEP at public clinics.

Importantly, our study found evidence that pharmacy-based PrEP delivery is not only in demand but is also, to some extent, already happening without official approval or oversight. This is not a new trend for retail pharmacies in Kenya, some of which have met early demand for products and services, such as HIV self-testing kits [36] and injectable contraceptives [18], by offering them before the Kenyan MOH granted explicit authorization to do so. Still, these reports of informal pharmacy-based PrEP delivery highlight the need for a more formalized care pathway that safeguards care quality and maximizes the public health impact of this intervention. Taking an implementation science approach, pharmacy PrEP implementers may be more likely to succeed if they pursue strategies that specifically target the determinants identified in our study [37, 38].

To address the determinant of intervention cost, strategies such as accessing new funding, developing resource-sharing agreements, and altering consumer fees may be necessary. Most pharmacy-based PrEP care in other countries, such as the U.S., is financed through private insurance or drug assistance programs sponsored by industry or the state that require little, if any, out-of-pocket payment from clients [5, 21]. In Kenya, clinic-based PrEP care is primarily funded by PEPFAR and the Global Fund [25]; retail pharmacy services are not covered by the national insurance scheme [39]; and less than 5% of the population has private insurance [40]. Additional research is, therefore, needed to explore possible financing mechanisms for pharmacy PrEP and to identify a price point that balances affordability for clients with the commercial interests of retail pharmacies.

Formalizing a pharmacy PrEP care pathway also entails re-negotiating pharmacy providers’ scope of practice. Currently, pharmacy providers in Kenya are allowed to carry out most, but not all, of the activities involved in PrEP delivery. For example, there is no legal provision in Kenya allowing retail pharmacy providers to prescribe PrEP or perform blood-based HIV testing, which is the only type of testing that the WHO currently recommends for initiating and continuing clients on PrEP [41, 42]. As such, successful implementation of pharmacy PrEP in Kenya will likely require strategies, such as revising pharmacy providers’ professional roles, conducting trainings, disseminating provider support tools, and providing expert consultation. Many U.S.-based pharmacy PrEP programs use formal agreements between PrEP prescribers and pharmacy providers known as “collaborative practice agreements” to give pharmacy providers special permission to carry out PrEP-related activities [5, 19, 21]. Kenya’s MOH could similarly decide to amend pharmacy practice to include PrEP delivery and work with the professional bodies for pharmacists and pharmaceutical technologists to integrate PrEP delivery competencies into continuing professional development education [43]. Participants in our study strongly supported the idea of establishing formal connections between pharmacy and PrEP providers who could provide expert consultation, when needed. Given that retail pharmacies and health clinics in Kenya are accustomed to operating independently, with little co-management of clients [44], pharmacy PrEP implementers may need to pursue various implementation strategies to enable inter-provider collaboration, such as establishing systems and protocols for provider communication and data sharing.

A final major consideration for implementers is quality assurance. Many participants in our study, including pharmacy providers, felt conflicted about for-profit PrEP services, anticipating, on the one hand, advantages over the free PrEP services in public clinics, but at the same time expressing doubt about “quacks” and “greedy” pharmacy providers who might prioritize money-making over client safety. Such concerns are not unfounded, as the Kenyan MOH has, in recent years, shut down hundreds of private pharmacies across Kenya that had expired or forged licenses, unqualified personnel, and/or counterfeit medicines [14, 45]. Our findings suggest that, for pharmacy PrEP to succeed, implementers will need to not only monitor care quality but also actively address client misgivings about pharmacy PrEP providers’ competency, professionalism, and motives. For the former task, implementers could consider whether and how existing quality monitoring tools [23] for clinic-based PrEP delivery might be integrated into the retail pharmacy setting. Instilling client confidence in pharmacy PrEP care quality will require a different set of strategies. For example, implementers could set up a system modeled after one established by the Kenyan MOH [46] whereby prospective PrEP clients can verify a provider’s “PrEP credentials” (e.g., nationally-certified PrEP training certificate) by sending a free SMS to a specified number.

Our study has some limitations. Participants’ perspectives on this hypothetical intervention may not accurately reflect how they would feel about it in a real-world scenario. We did not capture the perspectives of all pharmacy PrEP stakeholders, such as members of the Kenyan MOH. Because we did not interview adolescents, our findings may not reflect the perspectives of this priority population for PrEP. Lastly, we did not explore determinants of other implementation outcomes, like sustainability.

## Conclusions

For PrEP to impact the global HIV burden, sufficient coverage (i.e., uptake and persistence with sufficient adherence) must be achieved, especially among groups at high HIV risk. A one-size-fits-all delivery model is unlikely to reach the large, diverse, and geographically dispersed populations that could benefit from PrEP. If successful, Kenya’s pharmacy-based PrEP model could help differentiate PrEP care delivery and serve as a template for other ministries of health in sub-Saharan Africa to adapt. Future research is needed to develop and test tailored packages of implementation strategies that are most effective at integrating PrEP delivery into routine pharmacy practice in Kenya and other high HIV prevalence settings.

## Supplementary Information

Below is the link to the electronic supplementary material.

Supplementary file1 (DOCX 33 kb)

## Data Availability

Due to conditions of ethical approvals, we are unable to provide access to our full dataset on a public repository. However, we are willing to make partial de-identified transcripts available upon reasonable request. Interested persons should contact the corresponding author.
