# Supplementary material for: Acceptability and Feasibility of Pharmacy-Based Delivery of Pre-Exposure Prophylaxis in Kenya: A Qualitative Study of Client and Provider Perspectives
Source: AIDS Behav. 2021 Apr 7;25(12):3871–82. doi: 10.1007/s10461-021-03229-5 (PMC8602157; doi:10.1007/s10461-021-03229-5)
Supplement: Supplementary file 1 — Supplementary file1 (DOCX 33 kb) [file 10461_2021_3229_MOESM1_ESM.docx]

# Electronic Supplementary Material

*Additional details about study methodology*

| **Table S1**. Consolidated criteria for reporting qualitative studies (COREQ) checklist | | | |
| --- | --- | --- | --- |
| **Domain** | **No. Item** | **Guide Questions/Description** | **Section Where Reported** |
| Research Team and Reflexivity | ***Personal Characteristics*** | | |
|  | 1. Interviewer/facilitator | Trained research assistants conducted all interviews. | Methods |
|  | 1. Credentials | KFO has a ScD in Global Health and Population and a Master’s in Public Health. SR has a Master’s in Public Health and a BA in Cultural Anthropology. NW has a BA in Sociology. Other research assistants (RAs) who conducted interviews have university training in qualitative research and/or extensive prior field experience conducting qualitative interviews for HIV prevention research studies. | Title Page |
|  | 1. Occupation | Population Health Scientist (KFO), Research analyst/candidate for PhD in Global Health Implementation Science (SR), and research assistant (NW and other RAs who conducted interviews) | Not included |
|  | 1. Gender | Male and female | Not included |
|  | 1. Experience and training | KFO, SR, and NW have taken university-level courses in qualitative research. They and the other RAs who conducted interviews have prior experience collecting, analyzing, and publishing results from several qualitative research projects. | Methods |
|  | ***Relationship with Participants*** | | |
|  | 1. Relationship established | Relationships were limited to interviews. | Not included |
|  | 1. Participant knowledge of interviewer | Prior to interviews, RAs provided general descriptions of the study objectives, details of participation, and a brief personal introduction. | Methods |
|  | 1. Interviewer characteristics | No interviewer characteristics are reported. | Not included |
| Study Design | ***Theoretical Framework*** | | |
|  | 1. Methodological orientation and theory | Our approach is informed by Corbin and Strauss’s (2015) content analysis and Hsieh and Shannon’s refinement of it. In line with this approach, we collected data primarily through open-ended questions and derived our coding categories directly from the text data. | Methods |
|  | ***Participant Selection*** | | |
|  | 1. Sampling | We used purposive sampling to obtain participants from HIV clinics and retail pharmacies located in a variety of socioeconomic settings. Additional details not included in the methods section are that we purposefully sampled to have roughly equal numbers of men and women and clients above and below the age of 25 years in the two client samples. Also, we sampled PrEP clients across a range of durations of PrEP use and PrEP providers across a range of provider roles. | Methods |
|  | 1. Method of approach | RAs contacted pharmacy and PrEP providers in person or by phone to describe the research and invite them to participate. RAs approached potential client participants in person as they exited select pharmacies or HIV clinics and scheduled interested, eligible individuals for an interview. | Methods |
|  | 1. Sample size | 82 total interviews completed | Results |
|  | 1. Non-participation | Of 49 eligible pharmacy clients invited, 9 declined to participate due to time constraints. All other participant groups had 100% participation. | Results |
|  | ***Setting*** | | |
|  | 1. Setting of data collection | All interviews took place in a private room at a pharmacy, HIV clinic, or study research office. | Methods |
|  | 1. Presence of non-participants | Only the participant and interviewer were present during the interviews. | Not included |
|  | 1. Description of sample | Current adult pharmacy and PrEP clients and providers. | Methods, Results, and Table 1 |
|  | ***Data Collection*** | | |
|  | 1. Interview guide | We developed and pilot tested a de novo interview guide for each participant group. | Methods |
|  | 1. Repeat interviews | Each participant was interviewed once (i.e., no repeat interviews). | Methods |
|  | 1. Audio/visual recording | We audio-recorded all interviews with participant consent. | Methods |
|  | 1. Field notes | We did not collect field notes for this study. | Not included |
|  | 1. Duration | Each interview lasted approximately one hour. | Methods |
|  | 1. Data saturation | We did not conduct interviews to the point of data saturation. | Not included |
|  | 1. Transcripts returned | We did not return transcripts to participants for comment or correction. | Not included |
| Analysis and Findings | ***Data Analysis*** |  |  |
|  | 1. Number of data coders | Three: KFO, SR, and NW. | Methods |
|  | 1. Description of the coding tree | We provide a general description of how we first developed codes inductively and then used second cycle pattern coding to map our findings onto the Consolidated Framework for Implementation Research. We do not include a coding tree. | Not included |
|  | 1. Derivation of themes | Our reported themes emerged directly from the text data. | Methods and Results |
|  | 1. Software | Dedoose version 8.0.35 | Methods |
|  | 1. Participant checking | We did not provide participants with feedback on our findings. | Not included |
|  | ***Reporting*** | | |
|  | 1. Quotations presented | We present participant quotes illustrating our findings. Each quote is accompanied by a participant number. | Tables 2, 3, and 4 |
|  | 1. Data and findings consistent | Yes | Results |
|  | 1. Clarity of major themes | Our three major themes are identified by subheadings within the Results section. We discuss the relevance of these themes in the Discussion section. | Results and Discussion |
|  | 1. Clarity of minor themes | The specific determinants of pharmacy PrEP acceptability and feasibility are akin to minor themes. We discuss these in detail in the Results and Discussion sections. | Results and Discussion |

To ensure participants anchored their responses to the same understanding of PrEP and pharmacy-based PrEP delivery, interviewers presented participants with the following standard description of PrEP and pharmacy-based PrEP delivery prior to soliciting their perspectives:

| **Pharmacy PrEP description:**  I’d now like to shift topics to discuss a medication called pre-exposure prophylaxis or PrEP, for short. Have you ever heard of PrEP before? ***[Pause for answer. Then proceed.]***  As you may know, in 2015, the Kenyan Ministry of Health approved the use of PrEP for HIV prevention. If taken consistently every day, PrEP is a safe and effective way for individuals not living with HIV to reduce their risk of acquiring HIV. Currently, PrEP is only being delivered in select healthcare clinics in Kenya.  One of the objectives of this study is to understand **whether and how** PrEP might be delivered in private pharmacies, such as the one where you work.  At a minimum, in a pharmacy-based PrEP program, pharmacy staff would do the following:  ***[GIVE PARTICIPANT HANDOUT AND READ ALOUD]***:   - HIV testing - Counseling on HIV risk reduction strategies and PrEP, including the risks and benefits and importance of adherence - Prescribing PrEP with remote physician oversight (likely using a checklist to assess eligibility, with items such as *“Does the client know the HIV status of his/her sexual partner(s)?”*) - Dispensing and refilling PrEP - Assessing adherence - Screening for PrEP side effects and symptoms of HIV infection |
| --- |

| **Table S2. Characteristics of provider participant workplaces** | | | |
| --- | --- | --- | --- |
| **Variable** | **Health Clinics**^a^  **(n=9)** | **Pharmacies**  **(n=16)** |  |
| Sector |  |  |  |
| Public | 9 (100%) | 0 (0%) |  |
| Private | 0 (0%) | 16 (100%) |  |
| County^b^ |  |  |  |
| Kiambu County | 9 (100%) | 10 (63%) |  |
| Kisumu County | 0 (0%) | 6 (38%) |  |
| Location |  |  |  |
| Urban area - informal settlement | 1 (11%) | 6 (38%) |  |
| Urban area - non-informal settlement | 2 (22%) | 3 (19%) |  |
| Peri-urban area | 3 (33%) | 4 (25%) |  |
| Rural area | 3 (33%) | 3 (19%) |  |
| Number of days open per week; median (IQR) | 5 (5-5) | 7 (6-7) |  |
| Number of hours open per day; median (IQR) | 8 (8-8) | 13 (12-13) |  |
| Number of private consultation rooms^b^ |  |  |  |
| None | 0 (0%) | 4 (25%) |  |
| 1 | 1 (11%) | 9 (56%) |  |
| 2 | 3 (33%) | 2 (13%) |  |
| 3 or more | 5 (56%) | 1 (6%) |  |
| HIV testing options available^c^ |  |  |  |
| None | 0 (0%) | 6 (38%) |  |
| Provider-assisted testing only | 9 (100%) | 3 (19%) |  |
| Self-testing only | 0 (0%) | 1 (6%) |  |
| Both provider-assisted and self-testing | 0 (0%) | 6 (38%) |  |
| ^a^ At 7 of the 9 health clinics, PrEP was delivered exclusively in the Comprehensive Care Clinic (CCC) where clients living with HIV receive care and treatment services. At one clinic, PrEP was additionally available in the Prevention of Mother-to-Child Transmission (PMTCT) Clinic, and at another clinic, PrEP was additionally available in the outpatient department on weekends and public holidays; ^b^ Within the department where PrEP is delivered (for health clinics) or within the pharmacy (for pharmacies); ^c^ “Provider-assisted testing” includes blood- and oral fluid-based HIV tests conducted and interpreted on site by a healthcare provider. “Self-testing” includes the sale of blood- or oral fluid-based self-testing kits to clients who conduct and interpret the test off site (e.g., at home). | | | |

**Table S3. Participant recommendations to ensure acceptability of pharmacy-based PrEP delivery**

| **Specific Determinant** | **Definition** | **Recommendation** | **Illustrative Quote** |
| --- | --- | --- | --- |
| Privacy | Whether pharmacy has sufficient space to ensure client privacy | - *MOH/implementers*: Consider only allowing PrEP in pharmacies that have a private space for seeing clients. - *Pharmacy PrEP providers*: Consider delivery mechanisms that can enhance client privacy, such as phone-based PrEP counseling for prospective clients. | “The chemist should have a private room where they can engage the clients on confidential issues.” *(Kiambu Pharmacy Client 12)*  “[I’d feel more comfortable asking for PrEP at a pharmacy] if they had a number you could call and discuss, and then pick [up the drugs from the pharmacy] without talking much [in-person at the pharmacy].” *(Kisumu Pharmacy Client 11)* |
| Competency | Whether pharmacy providers have adequate knowledge and skills to deliver PrEP properly | - *MOH/implementers:* Require pharmacy providers to undergo PrEP training. - *Pharmacy PrEP providers:* Display PrEP credentials where clients can see them. | “The government should intervene and make that everyone working in a chemist [delivering PrEP] is a professional.” *(Kiambu PrEP Client 2)*  “It is a must for them to advertise their [PrEP training] certificate.” *(Kiambu PrEP Client 13)* |
| Professionalism | Whether pharmacy providers maintain confidentiality, treat clients with respect, and prioritize client well-being over profit-making | - *MOH/implementers:* Incorporate skills for maintaining confidentiality into PrEP training for pharmacy providers. - *Pharmacy PrEP providers*: Reassure clients that their information will be kept confidential, especially before commencing HIV testing and risk assessment. | “I think they [pharmacy PrEP providers] should just be trained on confidentiality so they may also assure their clients that there is 100% confidentiality." *(Kiambu Pharmacy Provider 6)*  “I think they [pharmacy PrEP providers] should reassure the client that ‘Whatever we are going to discuss is private. No one is going to hear this.’ And they make sure no one is hearing about it. At least that will relieve anxiety and make someone comfortable to share [their information].” *(Kisumu Pharmacy Client 6)* |
| Rapport | Whether the pharmacy provider and client have an ongoing, close relationship | - *MOH/implementers*: Train pharmacy providers on rapport-building skills - *Pharmacy PrEP providers:* Consider ways to organize delivery such that PrEP clients can see the same provider each time (e.g., designate one pharmacy provider to deliver PrEP; give clients the option to book an appointment); to increase client comfort discussing sexual activity, have clients fill out a questionnaire and use their responses to guide counseling. | “The people [PrEP providers] in the chemist, they should be well-versed on how to create rapport and create a friendly environment because the approach matters a lot.” *(Kiambu PrEP Client 14)*  “Some clients prefer they should have just that one person who should be taking care of their private health conditions. So maybe they should assign just one person in their chemist who should be taking care of such patients.” *(Kisumu Pharmacy Client 15)*  “Maybe if they have questionnaires…you can read through it by yourself and write their answers…[This would help clients who] can’t say it with their mouths.” *(Kiambu PrEP Client 4)* |
| Quality Regulations | Whether regulations instill confidence in quality of pharmacy PrEP care | - *MOH/implementers*: Only allow PrEP in registered pharmacies; designate entities responsible for monitoring pharmacy PrEP in pharmacies; establish a system for pharmacies to acquire PrEP commodities and a protocol for documenting PrEP dispensed and client information. | “The Pharmacy & Poisons Board can make sure that whichever people we have on the counter [at the pharmacy] are qualified personnel, in conjunction with the Kenya Pharmaceutical Association.” *(Kiambu Pharmacy Provider 2)*  “We have the SASCOs [Sub-County AIDS and STI Coordinators] who could do that [monitor the pharmacies] at the sub-county or county level.” *(Kiambu PrEP Provider 4)*  “Maybe we [the PrEP clinic] give [the pharmacy] 100 doses [of PrEP]. The pharmacy should always have the list of [PrEP] patients and [record] when it [dispensing] was done and when the client is due for their next refill—for the accountability of drugs.” *(Kiambu PrEP Provider 9)*  “[Pharmacy PrEP providers must have] adequate training and supervision…[The government must] follow up with those pharmacy providers to see, ‘Are they able to deliver it in the right way?’” *(Kiambu PrEP Provider 10)* |
| Affordability | Whether at-risk clients can afford pharmacy PrEP | *-MOH/implementers*: Consider the possibility of standardizing the price of pharmacy PrEP across pharmacies; consider subsidizing pharmacy PrEP to increase affordability to clients. | “The pharmacies should not determine [the price]. The Ministry [of Health] should put the price because you will find someone [some pharmacy] selling the way they want [i.e., at the price they want] such that people [in need of PrEP] will not benefit.” *(Kiambu Pharmacy Provider 9)*  “About the pricing, I think the Ministry [of Health] should come up with a fixed price [for PrEP] the same way it happens in hospitals … [Then,] the Ministry of Health should sensitize the public that the [PrEP] drugs can be accessed through the pharmacy but you will be charged for those services.” *(Kisumu Pharmacy Provider 4)*  “Maybe the government can cost share and kind of sponsor clients to get this service.” *(Kiambu Pharmacy Provider 3)*  “The government should be sponsoring this [pharmacy PrEP], just like it has done for HIV and AIDS drugs.” *(Kisumu Pharmacy Client 13)* |
| Profitability | Whether PrEP generates sufficient revenue for pharmacies | *-MOH/implementers*: If setting a standard price that pharmacy PrEP providers can charge, take into consideration the full range of costs incurred by pharmacies. | “[We would be doing] testing, counseling, and dispensing… and remember that the Public Health [Department] charges per kilogram of waste product. So disposing unused drugs will also come with the cost of offering the service.” *(Kiambu Pharmacy Provider 3)* |

**Table S4. Participant recommendations to ensure feasibility of pharmacy-based PrEP delivery**

| **Specific Determinant** | **Definition** | **Recommendations** | **Illustrative quote** |
| --- | --- | --- | --- |
| Space | Whether pharmacy can ensure client privacy | -*MOH/implementers*: Consider whether a private space should be a prerequisite for pharmacies delivering PrEP. | “You cannot answer such questions [about your sexual activity] at the [pharmacy] counter. There must be a place … a private room, where they [pharmacy providers] can engage clients on a bit of confidential issues.” *(Kisumu Pharmacy Client 19)* |
| Human Resources | Whether pharmacy has sufficient staff | - *Pharmacy PrEP providers:* Assess whether hiring additional staff is necessary; recommend that PrEP clients come during certain days/time of day when client volume is low. | “I will see how it will be working. If we don’t have enough time [to serve PrEP clients], I think we’ll have to add another employee.” *(Kiambu Pharmacy Provider 1)*  “I would give them [PrEP clients] a specific time, like, ‘Come around 10 A.M. There are usually no customers here then.’” *(Kiambu PrEP Provider 5)*  “[I would have PrEP clients come] in the morning hours because we’ll have enough time to deal with the client then … In the evening, it’s a bit hectic, so that person might be forced to wait for long … So we should be specific on the time [we suggest PrEP clients come].” *(Kisumu Pharmacy Provider 1)* |
| Supplies | Whether pharmacy has consistent access to supplies | - *MOH/implementers:* Establish reliable supply chains, especially for PrEP drugs and HIV testing kits; provide pharmacies with educational materials to give to current and prospective PrEP clients; consider giving pharmacies some supplies for free to keep costs to clients low. | “They [pharmacy PrEP implementers] need to make them [PrEP drugs] available for the common distributors. They should not be lacking—at least not in a place like Thika [the largest town in Kiambu County].” *(Kiambu Pharmacy Provider 10)*  “The government…[should] provide the [PrEP] medication and those [HIV testing] strips [and ensure] that they don’t run out in the pharmacies.” *(Kiambu Pharmacy Provider 9)* |
| Cycle Time | Whether the time it takes to serve a PrEP client aligns with the pharmacy’s workflow | *(See Human Resources, above)* | *(See Human Resources, above).* |
| Documentation | Whether the pharmacy’s record-keeping system can support pharmacy PrEP documentation | *MOH/implementers*: Establish a protocol for documenting pharmacy PrEP care and tracking PrEP clients across delivery points. | “[It would be easier for pharmacy PrEP and PrEP providers to work together] if pharmacies had the same PrEP registers [we have at the hospital] where you write a lot of information about the client and then the file that you will use in seeing the client.” *(Kiambu PrEP Provider 1)* |
| Provider Support Tools | Whether pharmacy PrEP providers have access to tools that facilitate PrEP delivery | - *MOH/implementers*: Adapt existing PrEP provider support tools for pharmacy providers and create new tools where needed. | “I have seen a checklist for ART [antiretroviral therapy] management. We can get something similar to that [for pharmacy PrEP].” *(Kisumu Pharmacy Provider 6)*  “You know that form [for] assessing adherence [that] we use in the [public health] facilities? You can also have that one [in pharmacy PrEP] so that at least when they [PrEP clients] come [for refills], you can ask them a few questions … [so] you’ll know how they’re doing.” *(Kisumu Pharmacy Provider 1)*  “They could come up with a questionnaire just to guide you [and]…to standardize things so that it can be easier for you to go through it very fast with clients. Then you can tell about their adherence.” *(Kiambu Pharmacy Provider 3)* |
| Competency | Whether pharmacy PrEP providers have adequate knowledge and skills to deliver PrEP properly | - *MOH/implementers:* Hold trainings, including regular CMEs, on PrEP. - *Pharmacy PrEP providers*: Consider hiring a clinician with PrEP expertise, | “One thing that should be put in place is a regular CME [continuous medical education]. Science is so dynamic. We need to be equipped with the day-to-day knowledge that comes up.” *(Kisumu Pharmacy Provider 5)*  “There should be a doctor [working at the pharmacy] who knows about the issues of [PrEP] drugs.” *(Kiambu PrEP Client 16)* |
| Networks of Care | Whether pharmacies and PrEP clinics are deliberately interconnected, thus allowing pharmacy PrEP providers to access PrEP expertise, as needed | - *MOH/implementers*: Establish connections between retail pharmacies and PrEP clinics and a protocol for consultations and referrals. | “[It would be good] for you to talk with those [PrEP] doctors and then … tell us, ‘This will be your [PrEP] doctor [you can consult] … In case there is a problem, please call this one.’” *(Kiambu Pharmacy Provider 8)*  “[Provider collaboration would work] if there’s someone who’s standing by so when there’s any issue, you can be able to contact them immediately and get an answer directly.” *(Kisumu Pharmacy Provider 1)*  “We should have a common shared database so you are able to track [clients] … with unique identifiers … You get to learn that, ‘Ok. Last time, they [the client] picked [PrEP] from this place. They were given drugs for 1 month.’ It even improves tracking adherence [because] you might see … they didn’t go back [for their refill] but they said they have been on continuous risk.” *(Kiambu PrEP Provider 10)* |
| Profitability | Whether PrEP generates sufficient revenue for pharmacies | - MOH/implementers: Consider subsidizing the cost of PrEP delivery to retail pharmacies. | “Being subsidized by the government in terms of the counseling costs [would help].” *(Kisumu Pharmacy Provider 3)*  “Whoever is sponsoring this [pharmacy PrEP] should also give [pharmacies] personnel to do it.” *(Kiambu Pharmacy Provider 6)* |
